# Early-Stage IM Treatment with the Host-Derived Immunostimulant CPDI-02 Increases Curative Protection of Healthy Outbred Mice Against Subcutaneous Infection with Community-Acquired Methicillin-Resistant *Staphylococcus aureus* USA300

**DOI:** 10.3390/pharmaceutics16121621

**Published:** 2024-12-21

**Authors:** Jason P. Stewart, Caleb M. Sandall, Jacob E. Parriott, Stephen M. Curran, Russell J. McCulloh, Donald R. Ronning, Joy A. Phillips, Robin Schroeder, Christy Neel, Kelly F. Lechtenberg, Samuel M. Cohen, Yazen Alnouti, Sohel Daria, D. David Smith, Joseph A. Vetro

**Affiliations:** 1 Department of Pharmaceutical Sciences, College of Pharmacy, University of Nebraska Medical Center, Omaha, NE 68198, USA; jason.stewart1@zoetis.com (J.P.S.); caleb.sandall@unmc.edu (C.M.S.); jacob.parriott@zoetis.com (J.E.P.); smcurran@unmc.edu (S.M.C.); don.ronning@unmc.edu (D.R.R.); yalnouti@unmc.edu (Y.A.); sdaria@unmc.edu (S.D.); 2 Department of Pediatrics, University of Nebraska Medical Center, Omaha, NE 68198, USA; russell.mcculloh@unmc.edu; 3 Biology Department, San Diego State University, San Diego, CA 92182, USA; jphillips@sdsu.edu; 4 Midwest Veterinary Services, Inc., Oakland, NE 68045, USA; robin@mvsinc.net (R.S.); christy@mvsinc.net (C.N.); kelly@mvsinc.net (K.F.L.); 5 Department of Pathology, Microbiology, and Immunology and the Buffett Cancer Center, University of Nebraska Medical Center, Omaha, NE 68198, USA; scohen@unmc.edu; 6 Department of Biomedical Sciences, Creighton University, Omaha, NE 68178, USA; 7 Center for Drug Delivery and Nanomedicine, University of Nebraska Medical Center, Omaha, NE 68198, USA

**Keywords:** antimicrobial resistance, AMR, antibiotic resistance, ABR, drug-resistant bacteria, antibiotic-resistant bacteria, multidrug resistance, MDR, host-directed therapy, EP67, complement peptide-derived immunostimulant, administration route, CPDI-02 toxicity, CPDI-02 dosing

## Abstract

**Background/Objectives**: Community-acquired methicillin-resistant *Staphylococcus aureus* (CA-MRSA) greatly complicates the treatment of skin and soft tissue infections (SSTI). It was previously found that subcutaneous (SQ) treatment with the mononuclear phagocyte (MP)-selective activator complements peptide-derived immunostimulant-02 (CPDI-02; formerly EP67) and increases prophylaxis of outbred CD-1 mice against SQ infection with CA-MRSA. Here, we determined if treatment with CPDI-02 also increases curative protection. **Methods**: Female CD-1 mice were challenged SQ with CA-MRSA USA300 LAC, then CPDI-02 or inactive scCPDI-02 was administered by a topical, SQ, IM, or IV route at 6 or 24 h post-challenge. Abscess sizes were compared over 10 days and CA-MRSA burden, neutrophils, MP, and pro-inflammatory cytokines were compared in subcutaneous abscesses. CPDI-02 PK and distribution in female CD-1 mice were compared after IM or IV dosing and CPDI-02 toxicity in male and female CD-1 mice was determined by IM dose escalation and repeat IM dosing. **Results**: Repeat IM treatment starting at 6 h post-challenge decreased maximum abscess surface area, CA-MRSA burden, and time to resolution, whereas repeat treatment by a topical, SQ, or IV route had no effect. Repeat treatment starting at 24 h post-challenge was ineffective by the current routes. Single IM treatment starting at 6 h post-challenge was as effective as repeat IM treatment, increased systemic exposure to CPDI-02, and, in subcutaneous abscesses, initially decreased IL-1β and increased MP. CPDI-02 was tolerated between 130 and 170 mg/kg after IM dose escalation and between 65 and 130 mg/kg after repeat IM dosing with males being more tolerant. **Conclusions**: Single early-stage IM treatment with CPDI-02 may increase curative protection against SSTI caused by CA-MRSA and/or other pathogens controlled by activated MP.

## 1. Introduction

Skin and soft tissue infections (SSTIs) of healthy skin (primary infection), damaged skin (secondary infection), and/or underlying fat, fascia, and muscle are the most common infections encountered in community or healthcare settings [1,2,3]. In the United States alone, 9.1 million cases of SSTI were diagnosed in 5.4 million patients from 2010 to 2020, with an average biennial recurrence of 26.3% and average annual treatment costs of USD 19.6 billion [4]. Over the same period, SSTIs were diagnosed in 1,065,444 emergency room visits and required 264,856 hospitalizations [4]. Worldwide, the age-standardized incidence of SSTI was estimated to be ~294 million in 2019 [5]. Thus, SSTIs are a tremendous global burden on healthcare infrastructure, resources, and costs.

*Staphylococcus aureus* (*S. aureus*) is a Gram-positive bacterium commonly found on the skin and mucous membranes of healthy individuals [6,7,8] that is one of the most frequently isolated pathogens from monomicrobial or polymicrobial SSTIs in the United States [1,6,9,10] and globally [10,11,12,13]. SSTIs that involve *S. aureus* can be acute, chronic, recurrent, or persistent [14], and potentially lead to a wide range of minor to life-threatening diseases such as cellulitis, primary and secondary abscesses, infected ulcers and wounds, toxic shock syndrome, catheter-associated infections, necrotizing fasciitis, and bacteremia [6,15].

Although *S. aureus* is sensitive to most antibiotics [16], its ability to quickly develop antibiotic resistance (ABR) through genomic mutations or horizontal transfer of ABR genes from other bacteria, and the widespread use of antibiotics [17], has led to the emergence of hospital-associated (HA) and community-associated (CA) strains of *S. aureus* with multidrug resistance (MDR) [6,11]. ABR strains of *S. aureus* were first reported in hospitalized patients shortly after the introduction of penicillin in 1942 [18], with >95% of isolates being penicillin-resistant today [17,19,20]. MDR strains of methicillin-resistant *S. aureus* (MRSA) were later reported in hospitalized patients (HA-MRSA) shortly after the introduction of methicillin in 1961 [20]. HA-MRSA strains then began to spread and evolve within community settings (CA-MRSA) in the 1990s and started correlating with a global increase in the incidence of SSTIs in 2000 [6,19]. MRSA strains have since become resistant to several other classes of antibiotics, including lincosamides, aminoglycosides, glycopeptides, macrolides, quinolones, oxazolidinones, and streptogramins [19,20], with frequencies and patterns of MDR varying by region over time [10]. This greatly complicates the empiric therapy of SSTI and requires the use of more toxic, broad-spectrum antibiotics that increase the risk of adverse events and more widespread MDR against these last-line treatments [21]. As such, given the relative risk of serious to fatal infections and the low rate of approval for new antibiotics, the US Centers for Disease Control (CDC) and the World Health Organization (WHO) have designated MRSA as a “serious threat” that is a “high priority” for increased development of novel antibiotics and therapeutic approaches to overcome MDR [13,22].

Complement Peptide-Derived Immunostimulant-02 (CPDI-02) (formerly EP67) is a novel, second-generation host-derived decapeptide agonist of Complement Peptide 5a (C5) Receptor 1 (C5aR1/C5a_1_R/CD88), which is based on the C-terminal pharmacophore of human Complement Peptide C5a (hC5a) [23,24,25]. Unlike hC5a (EC_50_ ~7 nM in primary human mononuclear phagocytes (MPs) and neutrophils (NPs) [26]), CPDI-02 minimizes potential NP-mediated toxicity by selectively activating MPs (monocytes, macrophages, dendritic cells) with 10^3^-fold greater potency than NPs (EC_50_ 167 nM in primary human MPs vs. 160 μM in primary human NPs) [26]. CPDI-02 is also immunostimulatory in mice [23,27,28,29,30,31,32,33], rabbits [34], and domestic pigs (unpublished). Given that activated MPs (especially monocytes and macrophages) broadly protect against most pathogens that cause monomicrobial and polymicrobial SSTI including Gram-positive bacteria, Gram-negative bacteria, and fungi [35,36,37,38], treatment with CPDI-02 alone or in combination with antimicrobials (“adjunct immunotherapy”) may improve the treatment of SSTIs caused by high-priority ESKAPE bacterial pathogens [39,40], like MRSA [41], and/or other pathogens, regardless of MDR status.

It was previously found that repeat subcutaneous (SQ) treatment of outbred mice with CPDI-02 before and after SQ challenge with CA-MRSA USA300 (three injections total) significantly decreased the maximum abscess surface area, the CA-MRSA load per gram of tissue, and the time to abscess resolution vs. inactive, scrambled scCPDI-02 [24]. Consistent with protection, SQ treatment with CPDI-02 increased local levels of IL-1β, IL-6, and CXCL1 and NP recruitment to the abscess [24]. In contrast, SQ treatment of C5aR1 knockout mice with CDPI-02 had no effect against SQ infection with CA-MRSA up to 48 h post-challenge vs. inactive scCPDI-02 [24], indicating that prophylactic SQ treatment with CPDI-02 increases protection against SQ infection with CA-MRSA through the activation of C5aR1. SSTIs, however, are treated in both prophylactic and curative settings [42]. Thus, it is important to determine if treatment with CPDI-02 also increases curative protection against SQ infection with CA-MRSA.

In this study, we determined (i) if CPDI-02 increases curative protection against SQ infection with CA-MRSA by comparing the effect of administration route, treatment time post-challenge, and dose frequency of CPDI-02 vs. inactive scrambled scCPDI-02 on maximum abscess surface area and time to resolution in female outbred CD-1 mice; (ii) if single IM treatment of female outbred CD-1 mice with CPDI-02 or scCDPI-02 at 6 h post-challenge is as effective as repeat IM treatment against SQ infection with CA-MRSA or affects levels of pro-inflammatory cytokines/chemokines, neutrophils, and/or mononuclear phagocytes in subcutaneous abscesses; (iii) if IM administration affects systemic exposure and distribution of CPDI-02 by comparing PK profiles and distributions of CPDI-02 after IM or IV administration in female outbred CD-1 mice; and (iv) preliminary toxicity of CPDI-02 after IM administration by IM dose escalation and repeat IM dosing in male and female CD-1 mice.

## 2. Materials and Methods

### 2.1. Large-Scale Synthesis of CPDI-02 and Inactive, Scrambled scCPDI-02

All reagents were from Sigma-Aldrich (St. Louis, MO, USA). Machine-assisted solid-phase peptide synthesis of CPDI-02 (Table 1) was performed on an AAPPTEC Apex 396 instrument (AAPPTec, Louisville, KY, USA) employing an eight-well reactor block. Assembly of CPDI-02 occurred on a 1 mmol scale on two aliquots of Fmoc-Arg(Pbf)-Wang resin (2 × 1.85 g, 0.27 mmol/g) in two separate parallel wells. Prior to assembly, each aliquot of resin was vortexed in DMF (14 mL) to swell the resin. After 30 min, the resin was filtered and vortexed with a 20% solution of piperidine in DMF twice (14 mL, 1 × 3 min, 1 × 9 min) to deprotect the Fmoc group. After washing with DMF (14 mL, 8 × 1 min), the resin was vortexed with a solution of Fmoc DAla-OH (0.62 g, 2 mmol) and HATU (0.76 g, 2 mmol) in DMF (12 mL) for 30 s. Next, 1 M DIEA in DMF (4 mL, 4 mmol) was added and the reaction mixture was vortexed for two hours. The resin was filtered and washed with DMF (14 mL, 5 × 1 min). This protocol was repeated using Fmoc-*N*MeLeu-OH, Fmoc-Pro-OH, Fmoc-Met-OH, Fmoc-Asp(*t*Bu)-OH, Fmoc-Lys(*t*Boc)-OH, Fmoc-Phe-OH, Fmoc-Ser(*t*Bu)-OH, and Fmoc-Tyr(*t*Bu)-OH to assemble CPDI-02. All coupling times were two hours, except for the addition of Fmoc-Pro-OH to the resin-bound *N*-methyl-leucyl-residue, which required 16 h for a negative chloranil test [43]. After deprotection of the final Fmoc group, the resulting peptide–resin was washed with DMF (14 mL, 5 × 1 min) and dichloromethane (14 mL, 5 × 1 min), and dried in vacuo. scCPDI-02 (Table 1) was assembled on a 0.5 mmol scale on Fmoc-Ser(*t*Bu)-Wang resin using the same protocols, with the exception that all couplings were complete within two hours.

For the cleavage of each peptide, a chilled cocktail of trifluoroacetic acid, phenol, water, and triisopropylsilane (88/5/5/2 *v*/*v*/*v*/*v*, 10 mL/g of peptide–resin) was added with stirring to the peptide–resin at room temperature. After two and a half hours, the resin was filtered, and crude peptide in the filtrate was precipitated in chilled diethyl ether, filtered, and lyophilized.

Crude peptides were purified by preparative reversed-phase high-performance liquid chromatography (RP-HPLC) on a Waters XSelect^®^ Prep CSH130 C18 column (50 × 250 mm, 5 μm, 130 Å pore size, catalog number 186007029) eluted at a flow rate of 100 mL/min using a solvent gradient of 0.25 N triethylammonium phosphate (TEAP, pH 2.25, Solvent A) and a mixture of the TEAP buffer and acetonitrile (2/3, *v*/*v*. Solvent B). Crude CPDI-02 (1.05 g) was dissolved in a mixture of Solvent A and Solvent B (77/23, *v*/*v*) and loaded onto the column, which had been previously equilibrated with the same solvent mixture. The product was eluted from the column by raising the content of Solvent B in the eluent to 43% over 75 min, and column effluent was continuously monitored at 214 nm and collected in fractions. Fractions containing the major peak were pooled, diluted two-fold with water, and loaded onto the same column now equilibrated with a mixture of 10 mM HCl (Solvent C) and acetonitrile (Solvent D) (95/5, *v*/*v*) at 100 mL/min. Raising the content of Solvent D in the eluent to 60% over 30 min eluted the product, which was collected in fractions. Lyophilization of pooled fractions containing ≥ 95% of the major product by RP-HPLC afforded the hydrochloride salt of CPDI-02 as a fluffy white powder (565 mg, yield 46%, [M + H]^+^ found 1241.652, theoretical 1241.635, Bruker autoflex maX). Crude scCPDI-02 was fractionated on the same column using a solvent gradient of 15%–85% Solvent B over 75 min, followed by desalting of the hydrochloride salt, as described previously, to yield the desired peptide as a fluffy white powder (232 mg, yield 37%, [M + H]^+^ found 1241.6315, theoretical 1241.6352, Thermo Q Exactive).

Peptide purity was determined by RP-UPLC using three different columns with the same gradient elution conditions: an ACQUITY BEH C18 column (150 × 2.1 mm, 1.7 μm, 130 Å pore size, catalog number 186003556), an ACQUITY BEH Phenyl column (150 × 2.1 mm, 1.7 μm, 130 Å pore size, catalog number 186003378), and an ACQUITY BEH C4 column (150 × 2.1 mm, 1.7 μm, 130 Å pore size, catalog number 186004497) from Waters (Milford, MA, USA) [26]. The solvents used were water containing 0.1% TFA (Solvent E) and acetonitrile containing 0.1% TFA (Solvent F). Peptides were eluted by increasing the percentage of Solvent F from 5 to 60% over 30 min at 0.2 mL/min and column effluent was continuously monitored at 214 nm. Both peptides were ≥95% pure on all three reversed-phase columns.

### 2.2. Topical Ointment Formulation of CPDI-02

For topical administration, CPDI-02 was formulated in an ointment of 3% (*w*/*v*) hydroxypropyl methylcellulose (HPMC) in 30% (*v*/*v*) propylene glycol [44] within 24 h of the first topical treatment. HPMC [270 mg] was added to propylene glycol [2.70 mL] at 100 mg HPMC/mL and mixed at 4 °C for 1 h while CPDI-02 [270 mg] was simultaneously dissolved in deionized water [6.5 mL] at 41.5 mg CPDI-02/mL. The CPDI-02 solution [6.3 mL] was added to the HPMC solution [2.4 mL] and mixed overnight to produce an ointment of 3% CPDI-02 (*w*/*v*) [3 mg/mL], aliquoted using a positive displacement pipette, and stored at 4 °C until use.

### 2.3. Bacterial Strain and Culturing

CA-MRSA USA300 LAC (*Staphylococcus aureus* Rosenbach, ATCC BAA-1756) was stored in 50% glycerol at −80 °C until use. To start a culture, a Tryptic Soy Agar (TSA; Remel) plate was streaked and incubated overnight at 37 °C. A single colony from the streaked agar plate was transferred to a 50 mL conical tube containing 30 mL of Tryptic Soy Broth (TSB; Remel) and incubated [37 °C] overnight while shaking [200 rpm]. The overnight culture was diluted [1:10] in TSB, incubated at 37 °C for ~2 h until A_600_ = 0.5 to 0.55, and pelleted [3000 g, 10 min] at 4 °C. The pellet was resuspended in PBS and pelleted as before and then resuspended in 0.05% (*w*/*v*) Cytodex-1 in sterile PBS (typical yield ~5 × 10^8^ CFU/mL) [45]. MRSA concentration was confirmed by serial dilution in sterile PBS, dot plating on TSA plates, and incubation at 37 °C for 24 h before counting.

### 2.4. Curative CPDI-02 Treatment of Healthy Female Outbred Mice Subcutaneously Infected with CA-MRSA

All procedures were approved by the University of Nebraska Medical Center Institutional Animal Care and Use Committee. Female outbred CD-1 mice (Charles Rivers, Strain Code 022, 4-6 weeks old) were acclimated in the animal facility for at least one week, and their rear flanks were shaved 24 h before the study. Mice were anesthetized with isoflurane and 0.100 mL of CA-MRSA stock solution (Section 2.3) was injected SQ (5 × 10^7^ CFU total) from the left rear flank into the dorsal side [45]. Repeat treatment: Vehicle alone or vehicle containing the indicated dose of CPDI-02 or inactive scCPDI-02 was administered (n = 10 mice) at 6 h post-challenge, then daily over 5 days starting at 24 h post-challenge (6, 24, 48, 72, 96, and 120 h post-challenge) by the indicated administration route (6 treatments total). For IM (left caudal muscle) and SQ (left flank) administration, CPDI-02 was administered in 0.050 mL of sterile PBS [20 mg/mL] (volume adjusted to 50 mg/kg dose). For topical administration (TOP), CPDI-02 was applied to abscesses in 0.100 mL of ointment (3% *w*/*v*) (Section 2.2) using a positive displacement pipette [150 mg/kg dose]. For IV administration (tail vein), CPDI-02 was administered in 0.050 mL sterile PBS [5 mg/mL] (volume adjusted to 12.5 mg/kg dose). Single IM treatment: At 6 h post-challenge, vehicle alone (PBS), or vehicle containing the indicated dose of CPDI-02 or inactive scrambled CPDI-02 (scCPDI-02), was administered (n = 10 mice) by the IM route as above (1 treatment total). Mouse abscesses were imaged with reference scales daily over 10 days starting at 24 h post-challenge. Abscess surface areas were determined by image analysis (ImageJ^®^, ver.1.54h) and compared between vehicle and treatment group for each administration route over ten days post-challenge by repeated measurement with two-way ANOVA with Geisser–Greenhouse correction.

### 2.5. CA-MRSA Burden of Subcutaneous Abscesses After Single IM Treatment with CPDI-02

At 24 h post-challenge (Section 2.4: single IM treatment), mice were euthanized (n = 5 per cohort) and rear flanks were disinfected with 70% EtOH. Abscess and skin biopsies (Tru-Punch Sterile Disposable Biopsy Punch Razors, 8 mm^2^, Sklar instruments 961130) were suspended in TSB [2 mL] and homogenized in C tubes (Miltenyi Biotec 130-093-237, Bergisch Gladbach, DE) using a gentleMACS Dissociator (Miltenyi Biotec 130-093-235, program B). Homogenized abscess tissues were serially diluted in PBS and plated on TSA plates and incubated at 37 °C for 24 h, and bacterial colonies were counted. Bacterial colony counts per abscess were normalized to starting tissue weights and compared using a Mann–Whitney test (nonparametric *t*-test). Sections of paraffin-embedded tissues collected above were also Gram-stained and analyzed on a Nikon Eclipse E600 (Minato City, Japan) at 200× magnification and imaged using a Lumenera Infinity 2 Microscope Camera (Ottawa, ON, Canada).

### 2.6. Proportions of Neutrophils and Mononuclear Phagocytes in Subcutaneous Abscesses After Single IM Treatment with CPDI-02

At 18 h post-treatment (24 h post-challenge) (Section 2.4: single IM treatment), mice were euthanized (n = 5 per cohort) and abscess and skin biopsies were collected (Section 2.5) and fixed in 10% formalin for 48 h. After tissue fixation, tissues were prepared into paraffin tissue sections by the Tissue Science Facility at the University of Nebraska Medical Center. Paraffin tissue sections were deparaffinated and immunostained for neutrophils (Ly6G) and mononuclear phagocytes (F4/80). Primary antibodies were Rat/IgG2a anti-F4/80 (14-4801-82 Invitrogen (BM8), 1:50) and Rat/IgG2b anti-Ly6 G/Ly6C (MA1-40038 Invitrogen (NIMP-R14), 1:50) diluted in TBS + 1% BSA, with overnight incubation. Secondary antibodies were Anti-Rat IgG2a—FITC (11-4817-82 Invitrogen, 1:200) and Anti-Rat IgG2b—eFluor 660 (50-4815-82 Invitrogen, 1:200), and both were incubated for 2 h. Sections were mounted on cover slides using 4’6’-diamidino-2-phenylindole (DAPI) and imaged on a Zeiss 710 Confocal Laser Scanning Microscope (Oberkochen, DE) under 200× magnification. Image files (.czi) were analyzed with ImageJ [46] using the following parameters. Import options: hyperstack/ROI manager; color mode: colorized/autoscale and split channels options checked; adjust/threshold/Otsu/red/dark background; process/binary/make binary; process/binary/fill holes; process/binary/watershed; analyze/analyze particles/size (micro^2^): 7 to 315 μm^2^/cicularity: 0.00 to 1.00/show: outlines/display results/clear results/summarize/exclude on edges/overlay. Average ratios ±SD of neutrophils (Ly6G^+^/Ly6C^+^) to nuclei (DAPI^+^), mononuclear phagocytes (F4/80^+^) to nuclei (DAPI^+^), and mononuclear phagocytes (F4/80^+^) to neutrophils (Ly6G/Ly6C^+^) in subcutaneous abscesses were compared by unpaired t test with Welch’s correction.

### 2.7. Pro-Inflammatory Cytokines and Chemokines in Subcutaneous Abscesses

At 3 h and 18 h post-treatment (Section 2.4: single IM treatment), mice were euthanized (n = 5 per cohort per time point), then abscess and skin biopsies were collected (Section 2.5), suspended in PBS [2 mL], and homogenized in C tubes (Miltenyi Biotec 130-093-237) using a gentleMACS Dissociator (Miltenyi Biotec 130-093-235, program B). Tissue homogenates were stored at −20 °C until analysis. Homogenized abscess tissues were then analyzed using a V-PLEX Pro-inflammatory Panel 1 Mouse Kit (Meso Scale Diagnostics K15048D-1) on a MESO QuickPlex SQ 120MM (MESO, Rockville, MD, USA) to measure the inflammatory markers of the abscesses.

### 2.8. Preliminary Comparison of CPDI-02 Systemic Exposure and Distribution After IM Administration to Outbred CD-1 Mice

Female outbred CD-1 mice (4-6 weeks old) were infected SQ with CA-MRSA, as described (Section 2.4). At 6 h post-infection, vehicle alone (PBS) or vehicle containing 12.5 mg CPDI-02/kg (IV) or 50 mg/kg (IM) and blood samples (50 µL) were collected from the submandibular vein (Medipoint Goldenrod Lancet, 5 mm) at 2, 4, 8, and 24 h post-treatment into EDTA-coated tubes containing 200 µL of acidified methanol (0.1% formic acid *v*/*v*), and immediately mixed by repeated inversion. Samples were pelleted (16,000 RCF) for 10 min, and supernatants were stored at −80 °C until analysis. At 24 h post-challenge, mice were euthanized, and rear flanks were disinfected with 70% ethanol. Abscesses (Section 2.5) and organs were excised, rinsed with deionized H_2_O, dried, and 50 mg sections were collected in 200 µL of acidified methanol, homogenized using a gentleMACS Dissociator (Miltenyi Biotec 130-093-235, Tokyo, Japan), and stored at −80 °C until analysis.

Peptide quantitation was performed using a Waters ACQUITY UPLC system coupled to a Xevo TQ-XS Tandem Quadrupole Mass Spectrometer with electrospray ionization. Samples (45 µL) were processed in EDTA tubes with 200 µL acidic methanol (0.1% formic acid), supplemented with 2.5 µL of sample solvent (1:1 MeOH:0.1% FA in H2O) and 2.5 µL of internal standard (IS: 20X scrambled CPDI-02, 1 µg/mL). Following pelleting (16,000 g, 10 min), 100 µL supernatant was diluted with 60 µL H_2_O prior to analysis. Chromatographic separation was achieved on an ACQUITY Premier Peptide^®^ BEH C18 Column (2.1 × 100 mm, 1.7 µm) at 25 °C. Mobile phases consisted of Buffer A: 7.5 mM ammonium formate (pH 3) and Buffer B: methanol with a flow rate of 0.25 mL/min over 10 min. The gradient profile was 20% B (0.0–1.0 min), 20-80% B (1.0–6.0 min), 80% B (6.0–7.0 min), 80-20% B (7–8 min), and 20% B (8–10 min). MS was operated in positive ESI mode with the following optimized parameters: cone gas (150 L/h), desolvation gas (900 L/h), collision gas (0.15 mL/min), capillary voltage (1.3 kV), cone voltage (42 V), source temperature (150 °C), and desolvation temperature (500 °C). The following MRM m/z values were monitored: 621.4 > 84 and 621.4 > 100 for the analyte and IS, respectively. Peptide concentrations were determined using analyte-to-IS peak area ratios. Calibration curves (0.1–1000 ng/mL) were generated in various matrices using a 1/y^2^ weighted least-squares linear regression with IS concentration maintained at 50 ng/mL.

### 2.9. Dose Escalation of CPDI-02 by the IM Route in Healthy Outbred CD-1 Mice

Twenty-four CD-1 mice, approximately 4 weeks old, were randomized by weight and sex into four cohorts and then intramuscularly administered either vehicle alone (filter-sterilized 0.2 μm endotoxin-free PBS) or vehicle containing CPDI-02 twice a week over 4 weeks (eight doses total). CPDI-02 doses began at 15 mg/kg and subsequent doses were increased by a conventional modified Fibonacci scheme (increase over previous dose: 100%, 67%, 50%, 40%, 30%, 33%, 33%) to a final dose of 225 mg/kg. Mice were weighed prior to the study and prior to each dose administration. On each dose administration date, mice were assigned a Pain and Distress Assessment Score (Table S1). Four days after the last treatment, mice were euthanized, whole blood was collected for complete blood count (CBC) with differential (VetScan^®^ HM5 Hematology Analyzer), and the thymus, heart, lungs, brain, liver, spleen, kidneys, femur bone marrow, popliteal lymph nodes, Peyer’s patches, and injection site muscle (left caudal thigh muscle) were collected. Collected tissues were fixed in 10% formalin for 48 h and then embedded in paraffin. Before embedment in paraffin, femur bone marrow samples underwent decalcification by shaking in 10% formic acid for 8 h. Paraffin samples were cut into sections and underwent a Hematoxylin and Eosin (H&E) stain, then analyzed for signs of inflammation and other tissue abnormalities utilizing a Nikon Eclipse E600 at 40× magnification, and imaged using a Lumenera Infinity 2 Microscope Camera.

### 2.10. Repeat Dosing of CPDI-02 by the IM Route in Healthy Outbred CD-1 Mice

Sixty CD-1 mice (thirty male and thirty female) approximately six weeks old were randomized into six cohorts (three cohorts for each gender) and then intramuscularly administered either vehicle alone (filter-sterilized 0.2 μm endotoxin-free PBS), vehicle containing CPDI-02 at 50% the observed MTD [65 mg/kg], or CPDI-02 suspended in PBS at 100% the observed MTD [130 mg/kg] twice a week over 32 days (nine doses total). Mice were weighed prior to each dose administration. On the day of administration, mice were assigned a Pain and Distress Assessment Score (Table S1). Four days after the last treatment, through terminal cardiac bleed, whole blood was collected for complete blood count (CBC) with differential (Abaxis VetScan^®^ HM5 Hematology Analyzer, Union City, CA, USA) and blood chemistry (Abaxis VetScan^®^ VS-2 Blood Chemistry Analyzer). In addition, injection site muscle tissues were collected, fixed in 10% formalin for 48 h, and then embedded in paraffin. Paraffin samples were cut into sections and underwent an H&E stain, then analyzed for signs of inflammation and other tissue abnormalities utilizing a Nikon Eclipse E600 (Nikon Corporation, Tokyo, Japan) at 200× magnification, and imaged using a Lumenera Infinity 2 Microscope Camera.

## 3. Results

### 3.1. Repeat Treatment with CPDI-02 Starting at an Early Stage of Subcutaneous Infection with CA-MRSA Increases Curative Protection of Healthy Female Outbred Mice Depending on the Route of Administration

**Figure 1:**
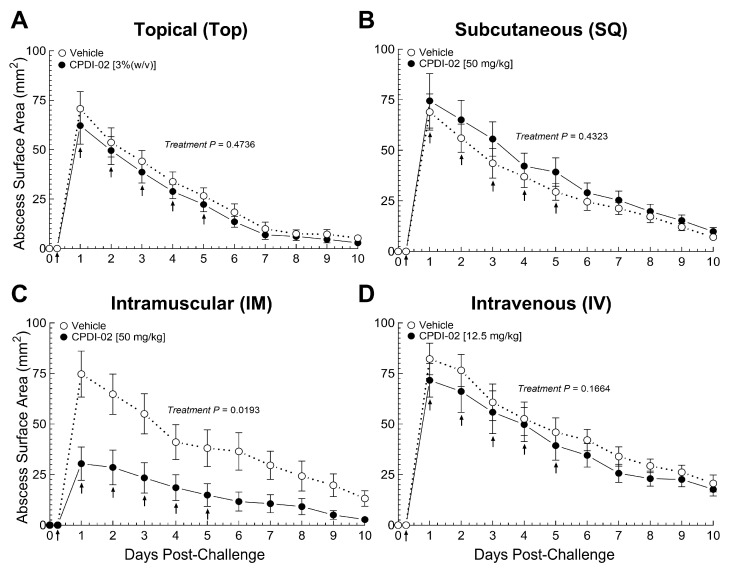
Repeat treatment with CPDI-02 increases curative protection of healthy female outbred mice against subcutaneous challenge with CA-MRSA depending on the route of administration. On Day 0, CA-MRSA (USA300 LAC, 5 × 10^7^ CFU) was injected SQ from the left rear flank into the dorsal side of 6-week-old female outbred CD-1 mice. Vehicle alone (white circles) or vehicle containing the indicated dose of CPDI-02 (black circles) was administered (↑) at 6 h post-challenge, then daily over 5 days starting 24 h post-challenge by the (**A**) topical (directly onto abscess), (**B**) subcutaneous (left flank), (**C**) intramuscular (left caudal thigh muscle), or (**D**) intravenous (tail vein) route. Average abscess surface areas ± SEM (n = 10 mice) from vehicle alone or vehicle containing CPDI-02 were determined daily by quantitative image analysis (Figure S1) and compared for each administration route by repeated measurement two-way ANOVA with Geisser–Greenhouse correction.

To establish that CPDI-02 increases curative protection against subcutaneous infection with CA-MRSA, we (i) injected CA-MRSA USA300 LAC subcutaneously into the rear dorsal side of healthy female outbred CD-1 mice [45], (ii) administered vehicle alone or vehicle containing CPDI-02 (Table 1) at the indicated dose by a topical (TOP), subcutaneous (SQ), intramuscular (IM), or intravenous (IV) route at 6 h post-challenge, then daily over 5 days post-challenge (6 treatments total), and (iii) compared surface areas of subcutaneous abscesses (Figure S1) daily over 10 days post-challenge by quantitative image analysis (Figure 1).

Compared to vehicle alone (Figure 1C, white circles), repeat treatment with 50 mg CPDI- 02/kg by the IM route (Figure 1C, black circles) decreased maximum abscess surface area (Day 1 post-challenge) by 58% [31 ± 8 (SEM) vs. 74 ± 11 mm^2^, *p* = 0.0052] and time to resolution by approximately 1 to 2 days. In contrast, repeat treatment with CPDI-02 by a TOP (Figure 1A, black circles), SQ (Figure 1B, black circles), or IV (Figure 1D, black circles) route had no effect on maximum abscess surface area or time to resolution vs. vehicle alone (Figure 1A,B,D, white circles). Repeat treatment with inactive scrambled scCPDI-02 (scCPDI-02; Table 1) [24] by the IM route at 50 mg/kg (Figure S2, black circles) also had no effect vs. vehicle alone (Figure S2, white circles), indicating that curative protection by IM treatment is due to the immunostimulatory activity of CPDI-02. Furthermore, repeat treatment with CPDI-02 starting at 24 h post-challenge had no effect, regardless of administration route. Thus, repeat treatment with CPDI-02 by an IM but not a TOP, SQ, or IV route increases curative protection against SQ infection with CA-MRSA when initiated early enough during infection under these experimental conditions.

### 3.2. Single Early-Stage IM Treatment with CPDI-02 Is Sufficient to Increase Curative Protection of Healthy Female Outbred Mice Against Subcutaneous Infection with CA-MRSA in a Dose-Dependent Manner

Initial IM treatment with CPDI-02 at 6 h post-challenge decreased maximum abscess surface area at 24 h post-challenge by 58% vs. vehicle alone (Figure 1C, Day 1 post-challenge, black vs. white circle). This suggested that subsequent IM treatments with CPDI-02 are not required to maintain the increase in curative protection against SQ infection with CA-MRSA.

**Figure 2:**
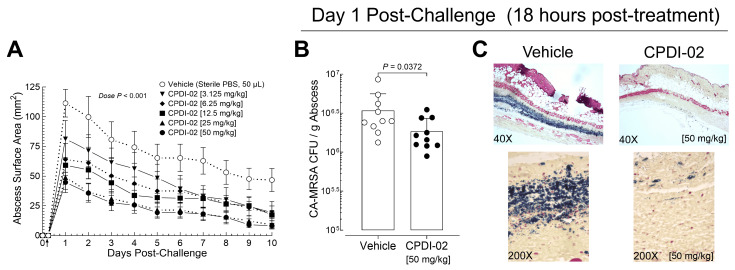
Single IM treatment with CPDI-02 is sufficient to increase curative protection of healthy female outbred mice against subcutaneous infection with CA-MRSA in a dose-dependent manner. On Day 0, CA-MRSA (USA300 strain, 5 × 10^7^ CFU) was administered SQ from the left rear flank into the dorsal side of 6-week-old female outbred CD-1 mice. (**A**) At 6 h post-challenge, vehicle alone (PBS, white circles) or vehicle containing the indicated dose of CPDI-02 (black symbols) was administered IM (↑) to the left caudal thigh muscle. Average abscess surface areas ± SEM (n = 10 mice) were then determined daily starting 24 h post-challenge by quantitative image analysis (Figure S1) and compared between doses by repeated measurement two-way ANOVA with Geisser–Greenhouse correction and Tukey’s post-test. On Day 1 post-challenge, (**B**) average colony forming units (CFU) of CA-MRSA/g of abscess biopsy ± SD (n = 10 mice) after treatment with vehicle alone (white circles) or vehicle containing CPDI-02 at 50 mg/kg (black circles) were determined by dilution plating and compared by two-tailed Mann–Whitney test and (**C**) the presence of CA-MRSA in subcutaneous abscess cross-sections was determined by histochemistry (HC) with Gram staining and imaging at 40× (2 mm wide) and 200× (400 μm wide) magnification. HC images are representative of 5 mice from each treatment group.

To determine if single early-stage IM treatment with CPDI-02 is sufficient to increase curative protection against subcutaneous infection with CA-MRSA, we (i) subcutaneously injected CA-MRSA USA300 LAC into the rear dorsal side of healthy female outbred CD-1 mice, (ii) administered vehicle alone or vehicle containing between 3.125 mg to 50 mg CPDI-02/kg by the IM route at 6 h post-challenge, (iii) compared abscess surface areas daily over 10 days post-challenge by quantitative image analysis (Figure 2A), and (iv) on Day 1 post-challenge, compared colony forming units (CFU) of CA-MRSA/g of subcutaneous abscess biopsies by dilution plating (Figure 2B) and the presence of CA-MRSA in subcutaneous abscesses by histochemistry (HC) with Gram staining (Figure 2C).

Single early-stage IM treatment with CPDI-02 (Figure 2A, black symbols) decreased maximum abscess surface area (Day 1 post-challenge) and time to resolution (Day 10 post-challenge) vs. vehicle alone (Figure 2A, white circles) as the dose of CPDI-02 increased with an ED_50_ of ~4 mg CDPI-02/kg. Single early-stage IM treatment with CPDI-02 at 50 mg/kg (Figure 2A, black circles) also decreased maximum abscess surface area (60% vs. 59% decrease on Day 1 post-challenge) and time to resolution to a similar extent as repeated early-stage IM treatment at the same dose (Figure 1C, black circles), despite the 50% larger maximum abscess surface areas in vehicle-treated mice vs. repeated IM treatment (Figure 2A vs. Figure 1C, black circles, Day 1 post-challenge) [111 ± 37 (SD) vs. 74 ± 36 mm^2^, *p* = 0.0433]. Single early-stage IM treatment with 50 mg CPDI-02/kg also decreased CA-MRSA CFU/g of abscess tissue on Day 1 post-challenge (Figure 2B, CPDI-02) by 1.9-fold vs. vehicle alone (Figure 2B, Vehicle) [1.8 × 10^6^ ± 0.8 × 10^6^ (SD) vs. 3 × 10^6^ ± 2 × 10^6^ CFU/g of abscess tissue] and qualitatively decreased detectable CA-MRSA in subcutaneous abscesses (Figure 2C, CPDI-02 vs. vehicle). Thus, single early-stage treatment with CPDI-02 by the IM route is sufficient to increase curative protection against subcutaneous infection with CA-MRSA in a dose-dependent manner under these experimental conditions.

### 3.3. Single Early-Stage IM Treatment with CPDI-02 Increases Mononuclear Phagocytes in Subcutaneous Abscesses of Female Outbred Mice During Subcutaneous Infection with CA-MRSA

**Figure 3:**
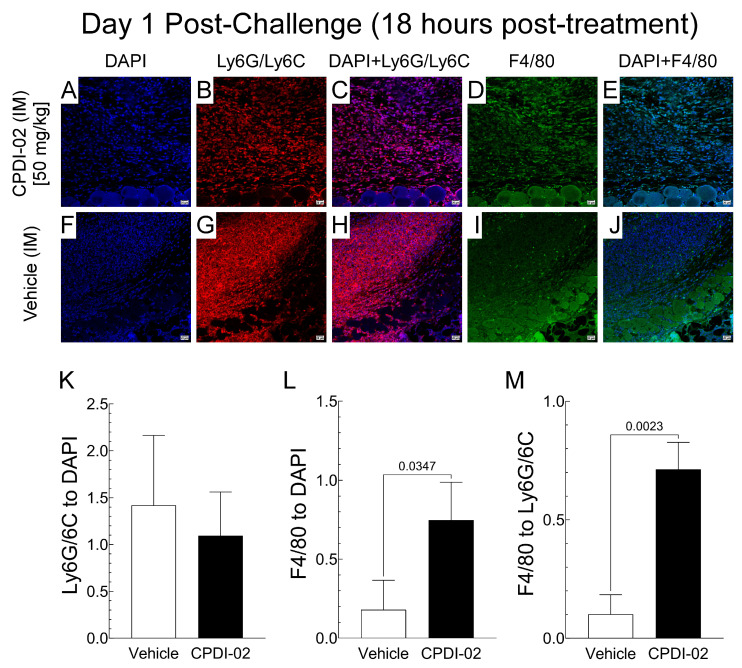
Single curative IM treatment with CPDI-02 increases the number of mononuclear phagocytes in subcutaneous abscesses from healthy female outbred mice at 18 h post-treatment. On Day 0, CA-MRSA (USA300 strain, 5 × 10^7^ CFU) was administered SQ from the left rear flank into the dorsal side of 6-week-old healthy female outbred CD-1 mice. At 6 h post-challenge, vehicle alone (PBS) or vehicle containing CPDI-02 at 50 mg/kg was administered IM to the left caudal thigh muscle. On Day 1 post-challenge (18 h post-treatment), the presence of (**A**,**F**;**C**,**H**;**E**,**J**) cell nuclei (DAPI^+^ cells, blue), (**B**,**G**;**C**,**H**) neutrophils (Ly6G/Ly6C^+^ cells, red), and (**D**,**I**;**E**,**J**) mononuclear phagocytes (F4/80^+^ cells, green) in subcutaneous abscesses was determined by IHC (200× magnification). Average ratios ±SD (n = 3 mice) of (**K**) neutrophils (Ly6G^+^/Ly6C^+^) to nuclei (DAPI^+^), (**L**) mononuclear phagocytes (F4/80^+^) to nuclei (DAPI^+^), and (**M**) mononuclear phagocytes (F4/80^+^) to neutrophils (Ly6G/Ly6C^+^) in subcutaneous abscesses were then determined by quantitative IHC image analysis and compared by unpaired t test with Welch’s correction. IHC images are representative of 3 mice from each treatment group.

To determine if single early-stage IM treatment with CPDI-02 affects the number of neutrophils (NP) and/or mononuclear phagocytes (MP) in subcutaneous abscesses after subcutaneous infection with CA-MRSA, we (i) subcutaneously injected CA-MRSA USA300 LAC into the rear dorsal side of healthy female outbred CD-1 mice, (ii) administered vehicle alone or vehicle containing 50 mg CPDI-02/kg by the IM route at 6 h post-challenge, and then (iii) compared ratios of NP (Ly6G/Ly6C^+^) and MP (F4/80^+^) to total cells (DAPI^+^) in subcutaneous abscesses by quantitative immunohistochemistry (IHC) image analysis at 18 h post-treatment (Figure 3).

Single early-stage IM treatment with CPDI-02 (Figure 3B,C) or vehicle alone (Figure 3G,H) had similar ratios of NPs (Ly6G/Ly6C^+^) to total cells (DAPI^+^) in subcutaneous abscesses (Figure 3K). In contrast, CPDI-02 (Figure 3D,E) increased ratios of MPs (F4/80^+^) to total cells (DAPI^+^) by 4-fold (Figure 3L) and mononuclear phagocytes to neutrophils (Ly6G/Ly6C^+^) by 7-fold (Figure 3M) vs. vehicle alone (Figure 3I,J). Thus, single early-stage IM treatment with CPDI-02 increases MPs in subcutaneous abscesses at an early stage of subcutaneous infection with CA-MRSA under these experimental conditions.

### 3.4. Single Early-Stage IM Treatment with CPDI-02 Decreases Early Levels of IL-1β in Subcutaneous Abscesses During Subcutaneous Infection with CA-MRSA

**Figure 4:**
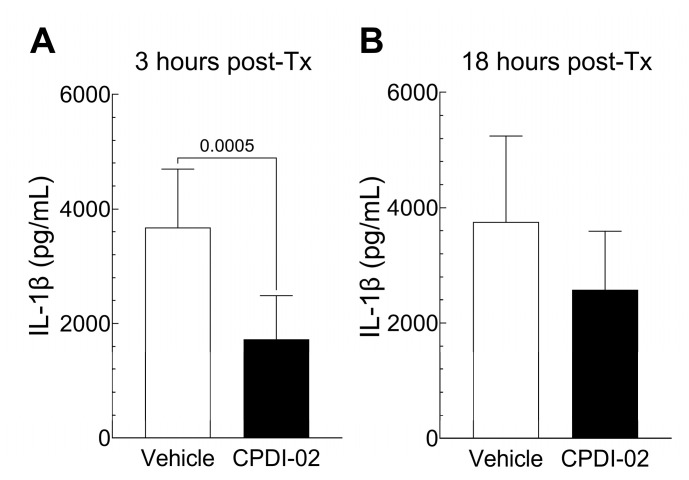
Single curative treatment with CPDI-02 by the IM route decreases early levels of IL-1β in subcutaneous abscesses of healthy female outbred mice after subcutaneous infection with CA-MRSA. On Day 0, CA-MRSA (USA300 strain, 5 × 10^7^ CFU) was administered SQ from the left rear flank into the dorsal side of 6-week-old healthy female outbred CD-1 mice. At 6 h post-challenge, vehicle alone (PBS, white bars) or vehicle containing CPDI-02 at 50 mg/kg (black bars) was administered IM to the left caudal thigh muscle. Average concentrations of pro-inflammatory markers in subcutaneous abscess biopsies ±SD (n=5 mice per time point) at (**A**) 3 h post-treatment (9 h post-challenge) or (**B**) 18 h post-treatment (24 h post-challenge) were determined by multiplex ELISA and compared by two-tailed t test with Mann–Whitney post-test (P-values shown). No differences in other major murine cytokines/chemokines involved in inflammation (IFN-γ, IL-2, IL-4, IL-5, IL-6, IL-10, IL-12p70, CXCL-1 [KC/GRO], TNF-α) were observed at these time points (Figure S3).

To determine if single early-stage IM treatment with CPDI-02 affects cytokines and chemokines potentially involved in local inflammation of subcutaneous abscesses after subcutaneous infection with CA-MRSA, we (i) subcutaneously injected CA-MRSA USA300 LAC into the rear dorsal side of healthy female outbred CD-1 mice, (ii) administered vehicle alone or vehicle containing 50 mg CPDI-02/kg by the IM route at 6 h post-challenge, and then (iii) compared major inflammatory cytokines and chemokines by multiplex ELISA (Figure 4 and Figure S3) at 3 h post-treatment (9 h post-challenge) or 18 h post-treatment (24 h post-challenge).

Single IM treatment with CPDI-02 at 6 h post-challenge decreased levels of IL-1β in subcutaneous abscesses at 3 h post-treatment (Figure 4A, black bar), but not 18 h post-treatment (Figure 4B, black bar), vs. vehicle alone (Figure 4, white bars) and had no effect on levels of IFN-γ, IL-2, IL-4, IL-5, IL-6, IL-10, IL-12p70, CXCL-1 [KC/GRO], or TNF-α at either time point (Figure S3). Thus, single early-stage IM treatment with CPDI-02 decreases early levels of IL-1β in subcutaneous abscesses after subcutaneous infection with CA-MRSA, but not most other cytokines/chemokines potentially involved in the inflammation of subcutaneous abscesses under these experimental conditions.

### 3.5. Comparison of IV and IM Administration on CPDI-02 Plasma Levels and Distribution to Organs and Subcutaneous Abscesses in Female Outbred Mice

**Figure 5:**
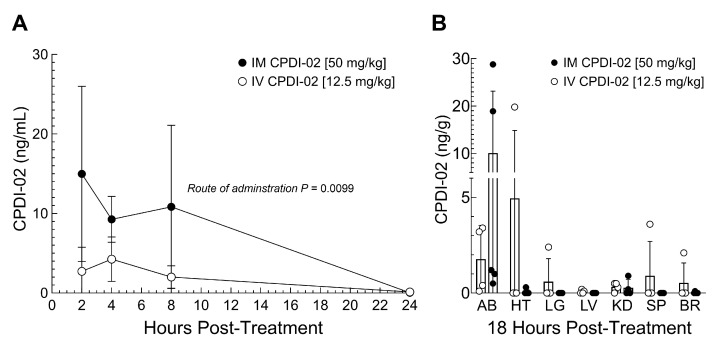
Preliminary PK and distribution of CPDI-02 in subcutaneous abscess-bearing female outbred mice after intravenous or intramuscular administration. On Day 0, CA-MRSA (USA300 strain, 5 × 10^7^ CFU) was administered SQ from the left rear flank into the dorsal side of 6-week-old healthy female outbred CD-1 mice. At 6 h post-challenge, vehicle (PBS, white circles) or vehicle containing CPDI-02 (black circles) was injected into the tail vein [12.5 mg/kg] (IV) or left caudal thigh muscle [50 mg/kg] (IM) of healthy female outbred CD-1 mice (6 weeks old), then (**A**) average plasma concentrations of CPDI-02 ± SD (n = 5 mice) over 24 h post-treatment, and (**B**) average masses of CPDI-02/g of abscess or indicated organ ±SD (n = 3 to 5 mice) at 24 h post-challenge were determined by LC-MS/MS and compared by two-way ANOVA. AB—abscess; HT—heart; LG—lungs; LV—liver; KD—kidneys; SP—spleen; BR—brain.

To determine if IM administration affects systemic exposure and/or distribution of CPDI-02 during subcutaneous infection with CA-MRSA, we (i) subcutaneously injected CA-MRSA USA300 LAC into the rear dorsal side of healthy female outbred CD-1 mice, (ii) administered vehicle alone or vehicle containing CPDI-02 by the IV [12.5 mg/kg] or IM [50 mg/kg] route at 6 h post-challenge, then (iii) compared the plasma concentrations of CPDI-02 at 2, 4, 8, and 24 h post-treatment (Figure 5A) and the normalized mass of CPDI-02 in organs and subcutaneous abscesses at 18 h post-treatment (24 h post-challenge) by LC-MS/MS (Figure 5B). We chose these IM and IV doses of CPDI-02, respectively, because repeat IM administration of 50 mg CPDI-02/kg to female outbred mice decreased maximum abscess surface area and time to resolution (Figure 1C), whereas repeat IV administration of 12.5 CPDI-02/kg had no effect (Figure 1A) vs. vehicle alone.

IM administration of CPDI-02 increased plasma concentrations (Figure 5A, black circles) vs. IV administration (Figure 5A, white circles) over 24 h, but had no effect on the relative distribution of CPDI-02 to the heart (HT), lungs (LG), liver (LV), kidneys (KD), spleen (SP), or brain (BR) at 18 h post-treatment (Figure 5B, black vs. white circles). IM administration also increased levels of CPDI-02 in a few subcutaneous abscesses (AB) (Figure 5B, AB, black circles) vs. IV administration (Figure 5B, AB, white circles) at 18 h post-treatment, but the overall difference between administration routes was just above statistical significance (*p* = 0.0636). Thus, single IM administration of CPDI-02 may increase curative protection against subcutaneous infection with CA-MRSA, in part, by increasing systemic exposure and subsequent localization of CPDI-02 to subcutaneous abscesses.

### 3.6. IM Dose Escalation of CPDI-02 in Healthy Male and Female Outbred Mice

**Figure 6:**
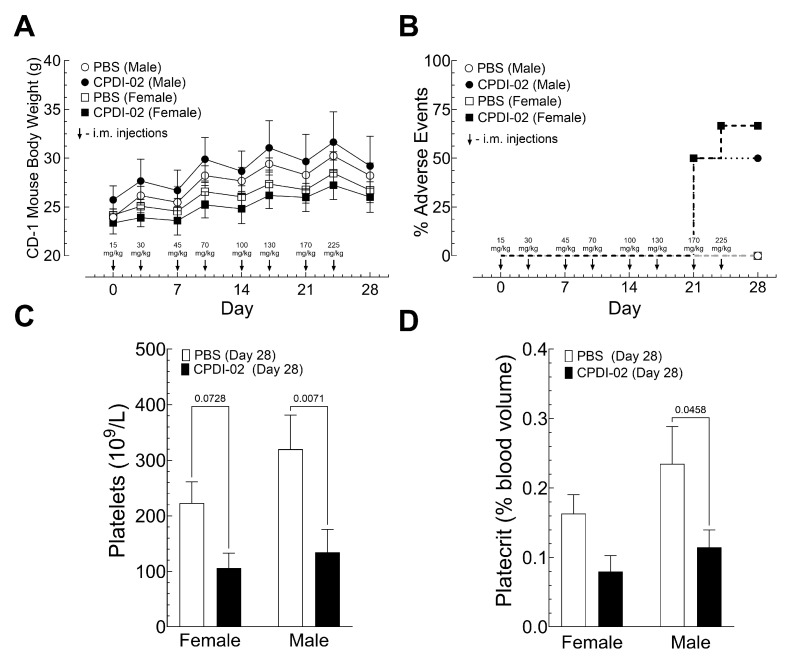
IM dose escalation of CPDI-02 in healthy male and female outbred CD-1 mice. (**A**) Vehicle alone (PBS, white symbols) or vehicle containing CPDI-02 (black symbols) was injected biweekly (↓) into the left caudal thigh muscle of healthy male and female outbred CD-1 mice (4 weeks old) at the indicated dose (black arrows) and average daily body weights ± SEM (n = 6 mice) were compared within each sex to vehicle alone by two-way ANOVA. (**B**) Pain and distress were scored on the day of each injection based on standardized signs in mice (Table S1). Signs of mild (score 2) to moderate (score 3) distress were observed starting 15 min after injection with CPDI-02 and resolved after 1 to 2 h. Four days after the final injection (Day 28), average values for complete blood count (CBC) with differential ±SD (n = 5 mice) were determined by hematology analyzer (Table S2) and compared by ordinary two-way ANOVA with uncorrected Fisher’s LSD and single pooled variance. Differences in CBC were only observed between (**C**) platelet concentrations and (**D**) platelet volumes.

To begin establishing a maximally tolerated dose (MTD) of CPDI-02 in outbred mice after IM administration, we (i) injected healthy male and female outbred CD-1 mice with vehicle alone or vehicle containing increasing doses of CPDI-02 up to 225 mg/kg in the left caudal thigh muscle twice per week over 28 days (8 injections total), then (ii) compared body weights by gravimetric analysis (Figure 6A) and pain and distress (Figure 6B) by single-blinded scoring assessment (Table S1) on the day of each injection, and (iii) compared complete blood count with differential by hematology analyzer (Figure 6C,D; Table S2) and signs of inflammation in tissues and organs by histochemistry with H&E staining (Figure S4) 4 days after the final injection. We started with ~4-week-old mice to ensure that later body weights would be low enough at the highest doses of CPDI-02 to accommodate the solubility of CPDI-02 in PBS (~100 mg/mL) in the maximum volume allowed for single IM injections in mice (0.05 mL).

IM dose escalation with PBS vehicle alone (Figure 6A, white circles [males] and squares [females]) or vehicle containing up to 225 mg CPDI-02/kg over 28 days (Figure 6A, black circles [males] and squares [females]) did not affect weight gain or the survival of healthy outbred CD-1 mice. IM administration of 170 or 225 mg CPDI-02/kg, however, increased signs of mild (Score 2) to moderate distress (Score 3) (Table S1) in male and female mice within 15 min of injection that resolved after 1 to 2 h (Figure 6B, black circles [males] and squares [females]).

Four days after the final dose (225 mg/kg) (Day 28), IM dose escalation with CPDI-02 decreased platelets in male and female mice (Figure 6C, black bars), decreased platecrit in male mice (Figure 6D, black bars) vs. vehicle alone (Figure 6C,D, white bars), and increased signs of inflammation in 83% of female mice and 50% of male mice (Figure S4B, black bars), whereas vehicle alone increased signs of inflammation around the injection site in 17% of female mice but not in male mice (Figure S4B, black bars). IM dose escalation with CPDI-02 or vehicle alone, however, did not increase signs of inflammation in the thymus, heart, lungs, brain, liver, spleen, kidneys, femur bone marrow, popliteal lymph nodes, or Peyer’s patches in male or female mice. Thus, IM administration of CPDI-02 is tolerated by healthy male and female outbred mice to somewhere between 130 and 170 mg CPDI-02/kg, with males likely tolerating a higher dose than females.

### 3.7. Repeat IM Dosing of CPDI-02 in Healthy Male and Female Outbred Mice

**Figure 7:**
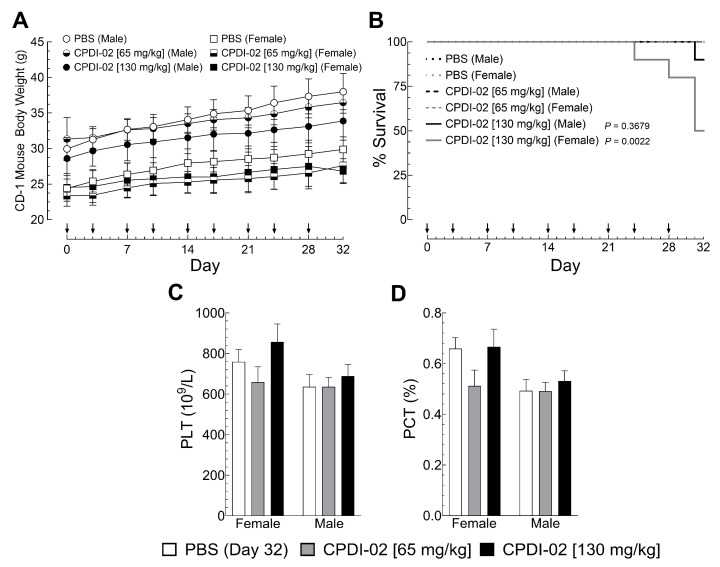
Repeat IM dosing of CPDI-02 in healthy male and female outbred mice. Vehicle alone (PBS) or vehicle containing CPDI-02 was injected biweekly (↓) into the left caudal muscle of healthy male and female outbred CD-1 mice (6 weeks old) at the indicated dose (black arrows) and (**A**) average daily body weights ± SEM (n = 10 mice) were compared within each sex to vehicle alone by two-way ANOVA and (**B**) survival for each treatment group was compared by simple Kaplan–Meier analysis with Mantel–Cox log-rank test (log-rank *p* values shown where relevant). No signs of distress were observed in any treatment group up to 2 h after injection. Four days after the final injection (Day 32), average values for complete blood count (CBC) with differential ±SD (Table S3) and analyte concentrations ±SD (Table S4) (n = 5 mice) were determined by a hematology and blood chemistry analyzer, respectively, and compared by ordinary two-way ANOVA with uncorrected Fisher’s LSD and single pooled variance. No differences in CBC were observed between (**C**) platelet concentrations and (**D**) platelet volumes.

To begin establishing the toxicity of CPDI-02 in healthy outbred mice after repeat IM dosing, we (i) injected healthy male and female outbred CD-1 mice with vehicle alone (endotoxin-free PBS) or vehicle containing CPDI-02 at 50% [65 mg/kg] or 100% of the observed MTD [130 mg/kg] (Section 3.6) in the left caudal thigh muscle twice per week over 32 days (9 injections total), then (ii) compared body weights by gravimetric analysis (Figure 7A) and pain and distress by single-blinded scoring assessment (Table S1) on the day of each injection, and (iii) compared complete blood count with differential by hematology analyzer (Table S3; Figure 7C,D), blood chemistry by blood chemistry analyzer (Table S4), and inflammation in tissues and organs by histochemistry with H&E staining (Figure S5) 4 days after the final injection.

Repeat IM dosing of vehicle alone (Figure 7A, white circles [males] and squares [females]) or vehicle containing 65 mg CPDI-02/kg (Figure 7A, black circles [males] and squares [females]) or 130 mg CPDI-02/kg (Figure 7A, black circles [males] and squares [females]) over 32 days had no effect on weight gain by healthy male or female outbred mice. Unlike dose escalation to 225 mg CPDI-02/kg (Figure 6B), repeat dosing with CPDI-02 did not increase signs of distress up to 2 h after injection or affect CBC (Table S3), including platelets (Figure 7C, gray and black bars) or platecrit (Figure 7D, gray and black bars), or blood chemistry (Table S4) in male or female outbred mice vs. vehicle alone (Figure 7C,D, white bars) up to 4 days after the final injection (Day 32). Repeat IM dosing with 130 mg CPDI-02/kg, however, increased mortality in female mice after the eighth and ninth IM injection (*p* = 0.0022) (Figure 7, gray line) but not in male mice (Figure 7, black line) (*p* = 0.3679) vs. vehicle alone. Repeat IM dosing with CPDI-02 also increased signs of mild to severe inflammation around the injection site compared to vehicle alone (Figure S5B, gray and black bars) in a dose-dependent manner and in a greater proportion of female mice than male mice at both 65 mg/kg and 130 mg/kg (Figure S5B, gray and black bars). There were, however, no signs of inflammation in the thymus, heart, lungs, brain, liver, spleen, kidneys, femur bone marrow, popliteal lymph nodes, or Peyer’s patches. Thus, repeat IM treatment with CPDI-02 is tolerated by healthy male and female outbred mice to somewhere between 65 and 130 mg CPDI-02/kg, with males likely tolerating a higher repeat dose than females.

## 4. Discussion

It was previously found that SQ treatment with the mononuclear phagocyte (MP)-selective activator, CPDI-02 (formerly EP67) [26], increases prophylaxis of outbred mice against SQ infection with CA-MRSA [24]. SSTI, however, are treated in both prophylactic and curative settings [42]. Thus, it is important to determine if treatment with CPDI-02 also increases curative protection against SQ infection with CA-MRSA.

The current study provides evidence that (i) repeat treatment with CPDI-02 by an IM route but not a TOP, SQ, or IV route increases curative protection against SQ infection with CA-MRSA when initiated early enough during infection under the current experimental conditions. We found that, compared to topical (3% (*w*/*v*)), SQ (50 mg/kg), or IV (12.5 mg/kg) treatment, repeat IM treatment with CPDI-02 (50 mg/kg) at 6 h post-challenge decreased maximum abscess size and time to resolution in female CD-1 mice after SQ infection with CA-MRSA USA300 LAC (Figure 1), whereas repeat IM treatment with inactive scrambled scCPDI-02 (Figure S2) or initiating repeat treatment with CPDI-02 at 24 h post-challenge had no effect vs. vehicle alone; (ii) single early-stage IM treatment with CPDI-02 increases curative protection against SQ infection with CA-MRSA to the same extent as repeat IM treatment and is dose-dependent. We found that single IM treatment of female outbred CD-1 mice with up to 50 mg CPDI-02/kg at 6 h post-challenge decreased maximum abscess surface area and time to resolution with increasing dose (ED_50_ ~4 mg CPDI-02/kg) (Figure 2) and, to a similar extent, as repeat IM treatment at the same dose [50 mg CPDI-02/kg] vs. vehicle alone (Figure 1); and (iii) CPDI-02 is tolerated by healthy outbred mice at supratherapeutic doses, with males likely tolerating higher doses than females. We found that IM dose escalation was tolerated by healthy male and female outbred CD-1 mice to somewhere between 130 and 170 mg CPDI-02/kg (Figure 6, Table S2, Figure S4), and repeat IM dosing was tolerated to somewhere between 65 and 130 mg CPDI-02/kg (Figure 7, Table S3, Figure S5). Furthermore, there were fewer histological signs of inflammation in male mice than female mice after dose escalation (Figure S4) and repeat dosing (Figure S5), and mortality was only observed in female mice after the eighth and ninth repeat IM injection at 130 mg CPDI-02/kg (Figure 7). In contrast, single early-stage IM treatment increased the protection of female outbred CD-1 mice with an ED_50_ of ~4 mg CPDI-02/kg (Figure 2).

Complement peptide 5a (C5a) (the parent molecule of CPDI-02 [26]) and its cleavage product C5a des-Arg [25] increase protection against bacterial pathogens at the site of infection by increasing activation of neutrophils and macrophages, acting as chemoattractants for neutrophils, monocytes, macrophages, and eosinophils, and inducing acute inflammation through local activation of mast cells, neutrophils, and endothelial cells [47]. During infection, local and plasma levels of C5a/C5a des-Arg are increased during the late steps of complement activation through the cleavage of C5 by C5 convertase assembled on the surface of microbes [47,48]. Thus, unlike topical, SQ, and IV administration, it is possible that early-stage IM treatment with CPDI-02 sufficiently increases CPDI-02 concentrations at the site of CA-MRSA infection, where it locally activates C5aR1-bearing cells before the late steps of complement activation in a manner similar to the parent molecule C5a (and C5a des-Arg) but with less toxicity associated with directly activating neutrophils and, possibly, mast cells. This mechanism is partially supported by our findings, as follows: single early-stage IM treatment with CPDI-02 (i) increased plasma concentrations of CPDI-02 over 24 h vs. IV administration of 15 mg CPDI-02/kg, whereas increases in local concentrations of CPDI-02 in subcutaneous abscesses vs. IV administration at 18 h post-treatment were just above statistical significance (*p* = 0.0636) (Figure 5), but may be more evident at earlier time points, and (ii) increased ratios of F4/80^+^ cell (mononuclear phagocytes), but not Ly6G/Ly6C^+^ cells (neutrophils), to DAPI^+^ cells (total cells) in abscess biopsies at 18 h post-treatment vs. vehicle alone (Figure 3), although NP may be increased in abscesses at earlier time points, as observed after prophylactic SQ treatment with CPDI-02 [24]. As such, given that abscess surface area roughly correlates with inoculum titers of CA-MRSA, our data suggest that early-stage IM treatment with CPDI-02 increases curative protection against SQ infection with CA-MRSA, in part, through sustained IM depot release/localization of CPDI-02 at the site of infection that increases recruitment and direct/indirect activation of mononuclear phagocytes, and, possibly, recruitment of neutrophils, and the subsequent rate that invasive CA-MRSA is cleared. Increasing the clearance rate of CA-MRSA could also decrease initial local concentrations of IL-β (Figure 4), given that CA-MRSA stimulates the secretion of IL-1β by neutrophils [49] and that neutrophils are the primary source of IL-1β in subcutaneous abscesses [50].

Interestingly, we found that, unlike repeat IM treatment, repeat SQ treatment with CPDI-02 at 50 mg/kg starting at 6 h *after* SQ challenge did not increase protection of female outbred CD-1 mice (Figure 1), whereas repeat SQ treatment with 12.5 mg CPDI-02/kg (formerly EP67) starting at 24 and 4 h *before* SQ challenge increases protection [24]. Given that CPDI-02 is expected to be cleared more quickly after SQ administration than IM administration, it is possible that curative treatment requires a more sustained localization of CPDI-02 at the site of infection than prophylactic treatment to sufficiently activate C5aR1-bearing cells. Furthermore, the reasons for increased toxicity of CPDI-02 in female vs. male outbred CD-1 mice after IM administration remain unclear but, given the role of mast cells (C5aR1-bearing cells [51]) in IgE-dependent and IgE-independent anaphylaxis [52], it might be related to sex-based differences in mast cell numbers and mast cell activation by CPDI-02 [53].

## 5. Conclusions

Single early-stage IM treatment with CPDI-02 increases curative protection of healthy outbred mice against SQ infection with CA-MRSA and may occur, in part, by increasing phagocytic activity at the site of infection. Thus, given that activated MPs (especially monocytes and macrophages) broadly protect against most pathogens that cause monomicrobial and polymicrobial SSTI, early-stage IM treatment with CPDI-02 alone or in combination with other antimicrobials (“adjunct immunotherapy”) may improve the prophylactic and curative treatment of SSTI caused by high-priority ESKAPE bacterial pathogens like MRSA and/or other pathogens, regardless of MDR status.

## Figures and Tables

**Table 1:** Molecular masses and sequences of CPDI-02 and inactive scrambled scCPDI-02.

Peptide	Molecular Mass (g/mol)	Amino Acid Sequence
CPDI-02	1241.6	H-Tyr^01^Ser^02^Phe^03^Lys^04^Asp^05^Met^06^Pro^07^NMeLeu^08^dAla^09^Arg^10^-OH
scCPDI-02	1241.6	H-NMeLeu^01^Arg^02^Met^03^Tyr^04^Lys^05^Pro^06^dAla^07^Phe^08^Asp^09^Ser^10^-OH

NMELeu = *N*-methyl-leucine.

## Data Availability

Data presented in this study are partially provided in the Supplementary Materials or available on request from the corresponding author.

## Supplementary Materials

The following supporting information can be downloaded at: https://www.mdpi.com/article/10.3390/pharmaceutics16121621/s1, Figure S1: representative images of subcutaneous abscesses on the rear dorsal region of CPDI-02-treated and untreated healthy female outbred mice 24 h after subcutaneous challenge with CA-MRSA; Figure S2: repeat IM treatment with inactive, scrambled CPDI-02 does not increase curative protection of healthy female outbred mice against subcutaneous challenge with CA-MRSA; Figure S3: single curative IM treatment with CPDI-02 does not affect early levels of many cytokines and chemokines potentially involved in the inflammation of subcutaneous abscesses after subcutaneous infection of healthy outbred female mice with CA-MRSA; Figure S4: histological comparison of inflammation around the injection site in left caudal thigh muscles from healthy male and female outbred mice after IM dose escalation of CPDI-02; Figure S5: histological comparison of inflammation around the injection site in left caudal thigh muscles from healthy male and female outbred mice after repeat IM dosing of CPDI-02; Table S1: pain and distress scoring in mice; Table S2: complete blood count with differential 4 days after IM dose escalation of CPDI-02 in healthy male and female outbred CD-1 mice; Table S3: complete blood count with differential 4 days after repeat dosing of CPDI-02 in healthy male and female outbred CD-1 mice; Table S4: blood chemistry 4 days after repeat IM dosing of CPDI-02 in healthy male and female outbred CD-1 mice.
