# Supplementary material for: Early-Stage IM Treatment with the Host-Derived Immunostimulant CPDI-02 Increases Curative Protection of Healthy Outbred Mice Against Subcutaneous Infection with Community-Acquired Methicillin-Resistant Staphylococcus aureus USA300
Source: Pharmaceutics. 2024 Dec 21;16(12):1621. doi: 10.3390/pharmaceutics16121621 (PMC11677424; doi:10.3390/pharmaceutics16121621)
Supplement: Supplementary file 1 [file pharmaceutics-16-01621-s001.zip › pharmaceutics-3331170-supplementary.pdf]

Supplementary Materials: **Early-stage IM treatment with the host-derived immunostimu-lant CPDI-02 increases curative protection of healthy outbred mice against subcutaneous infection with CA-MRSA USA300**

**Jason P. Stewart, Caleb M. Sandall, Jacob E. Parriott, Stephen M. Curran, Russell J. McCulloh, Donald R. Ronning, Joy A. Phillips, Robin Schroeder, Christy Neel, Kelly F. Lechtenberg, Samuel M. Cohen, Yazen Alnouti, Sohel Daria, D. David Smith and Joseph A. Vetro**

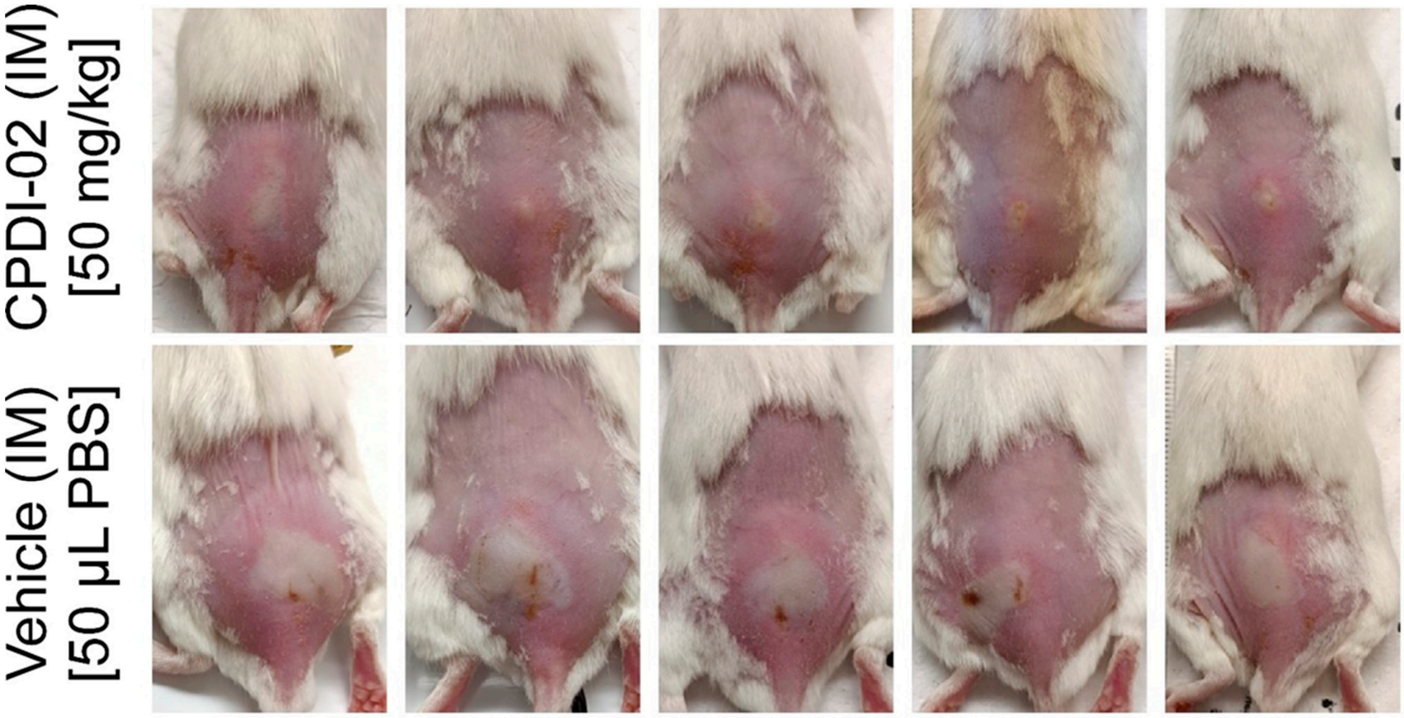


**Figure S1.** Representative images of dermal abscesses on the rear dorsal region of CPDI-02-treated and untreated healthy female outbred mice 24 hours after subcutaneous challenge with CA-MRSA. On Day 0, CA-MRSA (USA300 strain, $5\times10^7$ CFU) was administered SQ (0.1 mL sterile PBS) from the left rear flank into the dorsal side of 4 to 6-week-old healthy female outbred CD-1 mice (Figs.2&3). At 6 hours post-challenge, vehicle alone (sterile PBS, 50 µL) or vehicle containing CPDI-02 [50 mg/kg] was administered in the caudal thigh muscle and abscess surface areas (n=10 mice per cohort; 5 representative mice shown) were determined 24 hours post-challenge by image analysis.

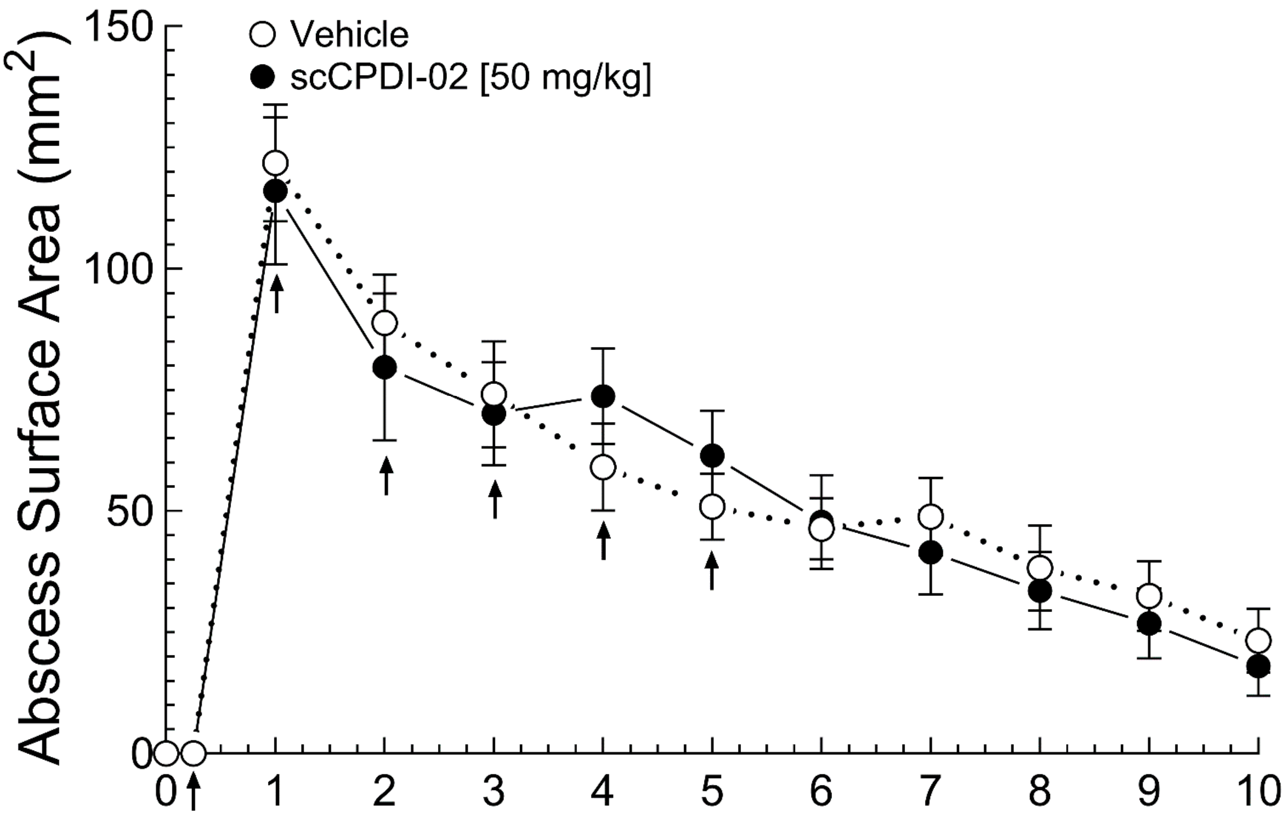


**Figure S2.** Repeat IM treatment with inactive, scrambled CPDI-02 does not increase curative protection of healthy female outbred mice against subcutaneous challenge with CA-MRSA. On Day 0, CA-MRSA (USA300 strain, $5\times10^7$ CFU) was administered SQ (0.1 mL sterile PBS) from the rear left flank into the dorsal side of 6-week-old female outbred CD-1 mice (**Fig.2**). At 6 hours post-challenge (↑), vehicle alone (50 μL sterile PBS, black circles) or vehicle containing 50 mg/kg inactive, scrambled CPDI-02 (scCPDI-02, white circles) was administered IM (left caudal thigh muscle). Average abscess surface areas ± SEM (n=10 mice) were determined daily starting 24 hours post-challenge by quantitative image analysis and compared between doses by repeated measurement 2-Way ANOVA with Geisser-Greenhouse correction and Tukey post-test

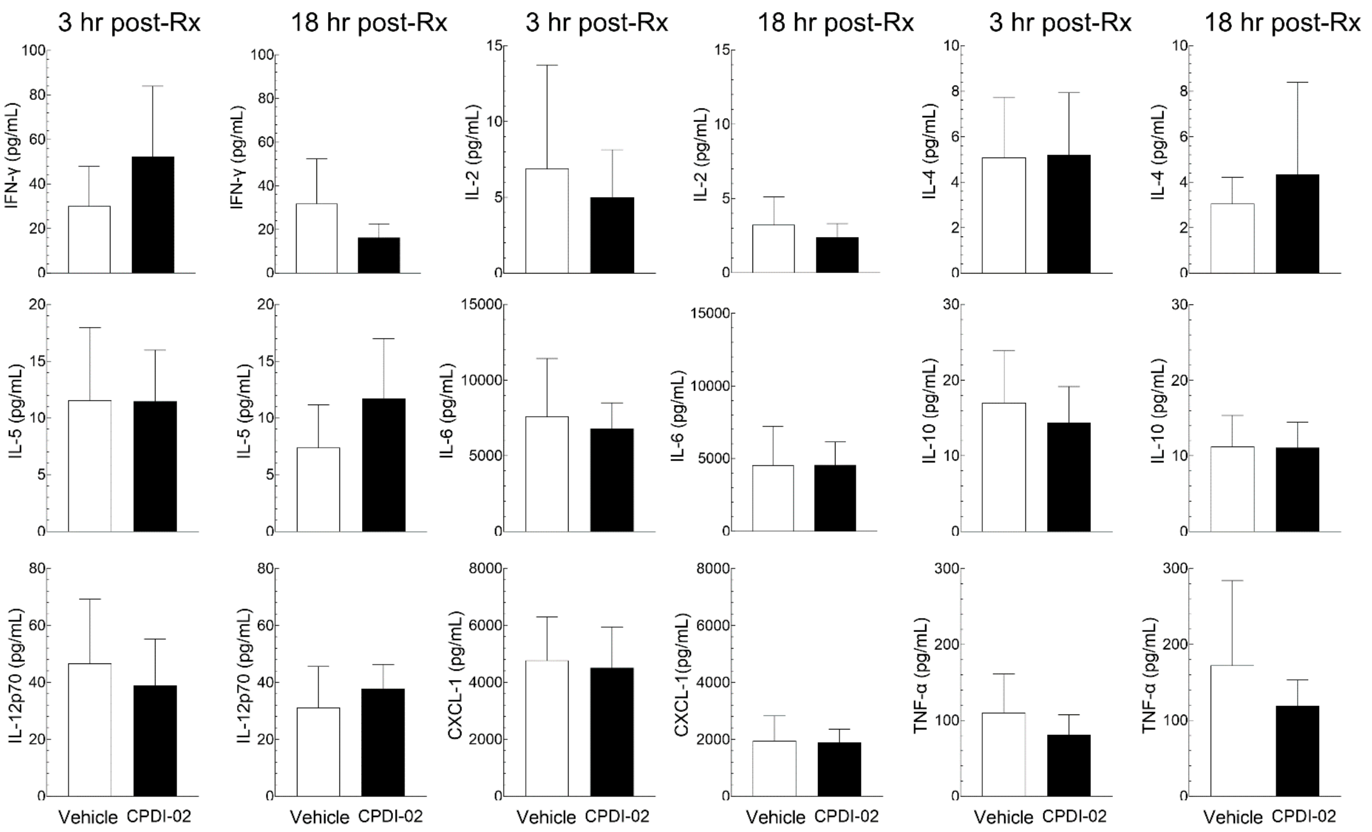


**Figure S3.** Single curative IM treatment with CPDI-02 does not affect early levels of many cytokines and chemokines potentially involved in the inflammation of subcutaneous abscesses after subcutaneous infection of healthy female outbred mice with CA-MRSA. On Day 0, CA-MRSA (USA300 strain, $5\times10^7$ CFU) was administered SQ (0.1 mL sterile PBS) from the left rear flank into the dorsal side of 6-week-old female outbred CD-1 mice. At 6 hours post-challenge, vehicle alone (white bars, sterile PBS, 50 μL) or vehicle containing CPDI-02 at 50 mg/kg (black symbols) was administered IM to the left caudal thigh muscle. Average concentrations of pro-inflammatory markers ±SD (n=5 mice per time point) in subcutaneous abscesses at (**A**) 3 hours post-treatment (9 hours post-challenge) or (**B**) 18 hours post-treatment (24 hours post-challenge) were then determined by multiplex ELISA and compared by two-tailed t test with Mann-Whitney post-test (P value shown)

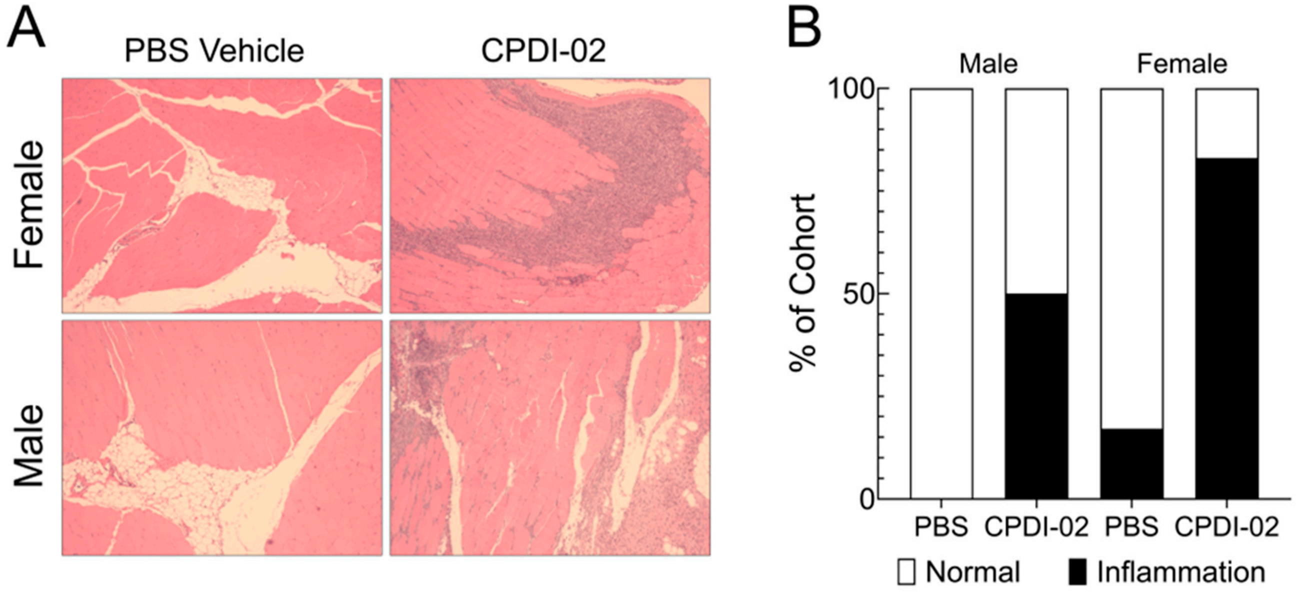


**Figure S4.** Histological comparison of inflammation around the injection site in caudal thigh muscles from healthy male and female outbred mice after IM dose escalation of CPDI-02. Vehicle alone (endotoxin-free PBS) or vehicle containing increasing doses of CPDI-02 was injected biweekly into the left caudal thigh muscle of healthy male and female outbred CD-1 mice over 28 days (**Fig.6**). Four days after the final injection, inflammation around the injection site was assessed by (**A**) histochemistry (HC) with H&E staining at 40X magnification (2 mm wide) and (**B**) proportion of each cohort with signs of inflammation was determined. HC images are representative of 10 mice from each treatment group

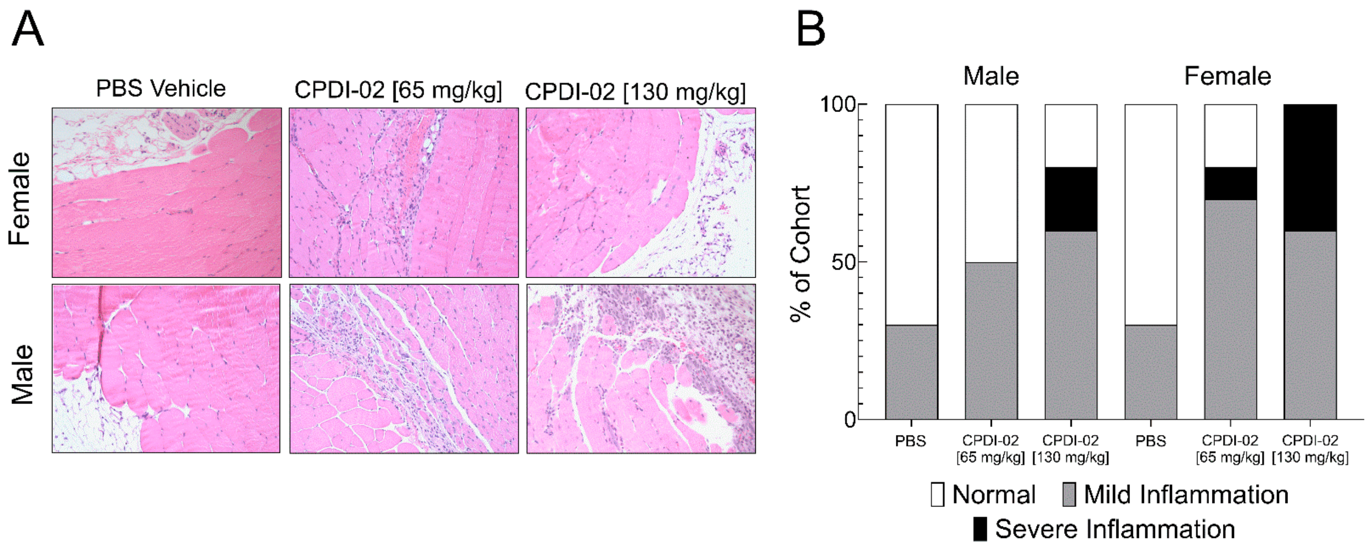


**Figure S5.** Histological comparison of inflammation around the injection site in caudal thigh muscles from male and female outbred mice after repeat IM dosing of CPDI-02. Vehicle alone (endotoxin-free PBS) or vehicle containing CPDI-02 (65 mg/kg or 130 mg/kg) was injected biweekly into the left caudal thigh muscle of healthy male and female outbred CD-1 mice over 28 days (**Fig.7**). Four days after the final injection, signs of inflammation around the injection site were assessed by (**A**) histochemistry (HC) with H&E staining at 40X magnification (2 mm wide) to determine (**B**) proportions of each cohort with signs of mild to severe inflammation. HC images are representative of 10 mice from each treatment group.

**Table S1 Pain and Distress Scoring in Mice**

| Score | Pain | Examples |
|---|---|---|
| 1 | No indication of pain or distress | Normal; well groomed; alert; active; good condition; asleep or calm; normal appetite |
| 2 | Mild or anticipated pain and distress | Not well groomed; awkward gait; slightly hunched; looks at injection site or pulls away when area is touched; mildly agitated |
| 3 | Moderate pain and distress | Rough hair coat; dirty incision; squinted eyes; moves slowly; walks hunched and/or slowly; depressed or moderately agitated; slight dehydration; pruritic; restless; uncomfortable; not eating or drinking. |
| 4 | Severe pain and distress | Very rough hair coat; eyes sunken (severe dehydration); slow to move or non-responsive when coaxed; hunched; large abdominal mass; dyspnea; self-mutilating; violent reaction to stimuli or when approached |

**Table S2 Complete Blood Count with Differential 4 Days after IM Dose Escalation of CPDI-02 in Healthy Male and Female Outbred CD-1 Mice**

| Analyte | Unit (±SD) | PBS (M) | PBS (F) | CPDI-02 (M) | CPDI-02 (F) |
|---|---|---|---|---|---|
| RBC Count | x10³/μL | 10 ± 2 | 10 ± 2 | 9 ± 1 | 9 ± 2 |
| Hematocrit | % | 52 ± 11 | 53 ± 10 | 45 ± 7 | 44 ± 9 |
| MCV | fL | 50 ± 3 | 54 ± 4 | 51 ± 3 | 51 ± 4 |
| MCH | pg | 15 ± 1 | 15 ± 2 | 15.4 ± 0.5 | 14.3 ± 0. 5 |
| MCHC | g/dL | 29 ± 3 | 27 ± 2 | 30 ± 2 | 28 ± 2 |
| RDW | % | 18.4 ± 0.9 | 19.1 ± 0.8 | 17.8 ± 0.5 | 18.6 ± 0.6 |
| Hemoglobin | g/dL | 15 ± 3 | 14 ± 3 | 13 ± 2 | 12 ± 3 |
| Platelet Count | x10³/μL | 320 ± 150 | 223 ± 93 | 135 ± 100 | 106 ± 66 |
| PCT | % | 0.2 ± 0.1 | 0.16 ± 0.07 | 0.11 ± 0.06 | 0.08 ± 0.06 |
| MPV | fL | 7.1 ± 0.8 | 7.4 ± 0.5 | 7 ± 1 | 7 ± 2 |
| PDW | fL | 11 ± 3 | 11 ± 3 | 11 ± 4 | 10 ± 2 |
| WBC Count | x10³/μL | 2.7 ± 0.8 | 3.4 ± 0.7 | 4 ± 1 | 3 ± 2 |
| Neutrophil Count | x10³/μL | 0.5 ± 0.3 | 0.5 ± 0.4 | 0.5 ± 0.5 | 0.2 ± 0.1 |
| Neutrophils | % | 16 ± 6 | 14 ± 10 | 15 ± 10 | 10 ± 6 |
| Monocyte Count | x10³/μL | 0.11 ± 0.05 | 0.2 ± 0.1 | 0.2 ± 0.1 | 0.13 ± 0.07 |
| Monocytes | % | 3.9 ± 0.7 | 6 ± 4 | 4 ± 2 | 5 ± 4 |
| Lymphocyte Count | x10³/μL | 2.1 ± 0.5 | 2.7 ± 0.5 | 3.1 ± 0.7 | 2 ± 2 |
| Lymphocytes | % | 80 ± 6 | 80 ± 13 | 80 ± 11 | 85 ± 9 |

Average complete blood cell count (CBC) with differential ±SD (n=5 mice) was determined four days after the final injection (**Fig.6**). (F) – female CD-1 mice; (M) – male CD-1 mice; MCV – mean corpuscular volume; MCH – mean corpuscular hemoglobin; MCHC – mean corpuscular hemoglobin concentration; RDW – red cell distribution width; PCT – plateletcrit; MPV – mean platelet volume; PDW – platelet distribution width; WBC – white blood cell.

**Table S3 Complete Blood Count with Differential 4 Days after Repeat IM Dosing of CPDI-02 in Healthy Male and Female Outbred CD-1 Mice**

| Analyte | Unit (±SD) | PBS (M) | PBS (F) | CPDI-02 65 mg/kg (M) | CPDI-02 65 mg/kg (F) | CPDI-02 130 mg/kg (M) | CPDI-02 130 mg/kg (F) |
|---|---|---|---|---|---|---|---|
| RBC Count | x10$^3$/μL | 9.9 ± 0.9 | 9 ± 3 | 10 ± 1 | 10 ± 3 | 11 ± 2 | 10 ± 2 |
| Hematocrit | % | 54 ± 6 | 46 ± 15 | 54 ± 7 | 54 ± 17 | 61 ± 12 | 55 ± 9 |
| MCV | fL | 55 ± 2 | 48 ± 1 | 55 ± 2 | 52 ± 2 | 54 ± 1 | 54 ± 2 |
| MCH | pg | 15.7 ± 0.6 | 14 ± 4 | 15.7 ± 0.5 | 15.6 ± 0.7 | 15.3 ± 0.4 | 16 ± 1 |
| MCHC | g/dL | 28.5 ± 0.9 | 30 ± 8 | 28 ± 1 | 30 ± 2 | 28.1 ± 0.9 | 29 ± 1 |
| RDW | % | 23 ± 1 | 21 ± 2 | 23 ± 1 | 24 ± 3 | 25 ± 2 | 24 ± 2 |
| Hemoglobin | g/dL | 15 ± 1 | 16 ± 5 | 15 ± 2 | 16 ± 4 | 17 ± 3 | 16 ± 2 |
| Platelet Count | x10$^3$/μL | 636 ± 188 | 759 ± 196 | 636 ± 146 | 659 ± 237 | 689 ± 170 | 857 ± 198 |
| PCT | % | 0.5 ± 0.1 | 0.6 ± 0.2 | 0.5 ± 0.1 | 0.5 ± 0.2 | 0.5 ± 0.1 | 0.7 ± 0.2 |
| MPV | fL | 7.8 ± 0.3 | 8.3 ± 0.3 | 7.7 ± 0.6 | 7.6 ± 0.6 | 7.7 ± 0.3 | 7.8 ± 0.3 |
| PDW | fL | 7.4 ± 0.3 | 7.3 ± 0.3 | 7.6 ± 0.6 | 7.8 ± 0.9 | 8.2 ± 0.7 | 7.4 ± 0.5 |
| WBC Count | x10$^3$/μL | 7 ± 2 | 7 ± 3 | 6 ± 2 | 7 ± 2 | 6 ± 2 | 8 ± 3 |
| Neutrophil Count | x10$^3$/μL | 2.6 ± 0.8 | 1.3 ± 0.7 | 2.6 ± 0.8 | 3 ± 1 | 2 ± 1 | 3 ± 1 |
| Neutrophils | % | 37 ± 5 | 20 ± 5 | 41 ± 10 | 30 ± 18 | 45 ± 17 | 33 ± 8 |
| Monocyte Count | x10$^3$/μL | 0.03 ± 0.01 | 0.02 ± 0.01 | 0.03 ± 0.01 | 0.02 ± 0.01 | 0.02 ± 0.02 | 0.03 ± 0.01 |
| Monocytes | % | 0.4 ± 0.2 | 0.2 ± 0.1 | 0.4 ± 0.2 | 0.3 ± 0.2 | 0.4 ± 0.2 | 0.3 ± 0.1 |
| Lymphocyte Count | x10$^3$/μL | 4 ± 1 | 5 ± 2 | 4 ± 1 | 5 ± 1 | 3 ± 2 | 5 ± 2 |
| Lymphocytes | % | 60 ± 6 | 77 ± 5 | 55 ± 11 | 62 ± 12 | 51 ± 17 | 63 ± 7 |

Average complete blood cell count (CBC) with differential ±SD (n=5 mice) was determined four days after the final injection (**Fig.7**). (F) – female CD-1 mice; (M) – male CD-1 mice; MCV – mean corpuscular volume; MCH – mean corpuscular hemoglobin; MCHC – mean corpuscular hemoglobin concentration; RDW – red cell distribution width; PCT – plateletcrit; MPV – mean platelet volume; PDW – platelet distribution width; WBC – white blood cell.

**Table S4 Blood Chemistry 4 Days after Repeat IM Dosing of CPDI-02 in Healthy Male and Female Outbred CD-1 Mice**

| Analyte | Unit (±SD) | PBS (M) | PBS (F) | CPDI-02 65 mg/kg (M) | CPDI-02 65 mg/kg (F) | CPDI-02 130 mg/kg (M) | CPDI-02 130 mg/kg (F) |
|---|---|---|---|---|---|---|---|
| ALB | g/L | 41 ± 2 | 42 ± 3 | 39 ± 2 | 41.1 ± 0.7 | 40 ± 2 | 41 ± 2 |
| ALP | U/L | 71 ± 22 | 86 ± 19 | 66 ± 18 | 74 ± 28 | 61 ± 13 | 77 ± 49 |
| ALT | U/L | 132 ± 285 | 136 ± 153 | 41 ± 24 | 39 ± 19 | 100 ± 134 | 200 ± 257 |
| AMY | U/L | 1005 ± 440 | 1199 ± 988 | 1032 ± 582 | 734 ± 169 | 867 ± 138 | 659 ± 62 |
| TBIL | μM | 4.1 ± 0.8 | 4.1 ± 0.8 | 4 ± 1 | 6 ± 1 | 4 ± 1 | 4.4 ± 0.8 |
| BUN | mM | 12 ± 1 | 10 ± 2 | 11 ± 1 | 10 ± 1 | 11 ± 1 | 9 ± 2 |
| CA | mM | 2.5 ± 0.1 | 2.31 ± 0.05 | 2.5 ± 0.2 | 2.4 ± 0.1 | 2.46 ± 0.07 | 2.3 ± 0.1 |
| PHOS | mM | 1.9 ± 0.4 | 3 ± 1 | 2.4 ± 0.5 | 2.6 ± 0.9 | 2.2 ± 0.4 | 2.7 ± 0.7 |
| GLU | mM | 15 ± 3 | 13 ± 4 | 17 ± 2 | 15 ± 3 | 13 ± 5 | 14 ± 4 |
| Na | mM | 151 ± 6 | 148 ± 3 | 152 ± 7 | 148 ± 4 | 147 ± 2 | 146 ± 2 |
| K | mM | 5 ± 1 | 5 ± 1 | 5 ± 1 | 4.1 ± 0.3 | 5.6 ± 0.8 | 5 ± 1 |
| TP | g/L | 51 ± 3 | 49 ± 4 | 50 ± 2 | 51 ± 4 | 50 ± 1 | 49 ± 3 |
| GLOB | g/L | 10 ± 2 | 7 ± 2 | 11 ± 2 | 9 ± 3 | 11 ± 1 | 7 ± 2 |

Average analyte concentration ±SD (n=5 mice) was determined four days after the final injection (**Fig.7**). (F) – female CD-1 mice; (M) – male CD-1 mice; ALB – albumin (kidney/liver); ALP – alkaline phosphatase (liver/bone); ALT – alanine transaminase (liver); AMY – amylase (pancreas); TBIL – bilirubin (liver); BUN – blood urea nitrogen (kidneys); CA – calcium (bone/thyroid/parathyroid/kidneys); PHOS – phosphate (kidney/bone/parathyroid); GLU – glucose (pancreas); Na – sodium; K – potassium; TP – total protein (liver/kidney); GLOB – globulin (liver/kidney).
